# Is Our Knowledge of Lunacy Stationary?

**Published:** 1896-09-19

**Authors:** 


### Is Our Knowledge of Lunacy Stationary?

It is a little surprising, and more than a little disappointing, to be told, regarding insane persons, as we have recently been told, that "On the questions of why and where the brain breaks down, of the earliest indications that it is in danger, or of the means which might be taken to fortify or secure it, medicine, so far, has scarcely advanced beyond the obvious teachings of common sense." Now we venture to affirm that this statement entirely and injuriously misrepresents the whole business. Insanity and insane persons have been studied by men as intelligent, as conscientious and as competent as any who have pursued other special departments in medicine. Not only so, but the results, especially if we take them in the mass, will be found to be as striking and as beneficent as any which can be shown by any other department in general or special practice. Of those results nothing more need be said at present than this, that whereas a hundred years ago the savages who "clubbed" their helpless fellows treated them with more humanity and common sense than civilisation showed to its lunatics, today intelligence, science and benevolence combine to take from their lot every unhappiness which is by any possibility removable, and to add to that ot every happiness, every comfort, every help which can by any possibility be devised. It is in the mass, after all, that general questions must mainly be considered; and such is the progress and such are the results of the study of lunacy in the mass by the medicine of the present century.

But in detail, as well as in the mass, there is everywhere evident the same advance from crudeness to science, from brute ignorance to comprehension, from savage repression to intelligent curative effort. But the question must be considered philosophically. The natural difficulties of the subject must be perceived and admitted; and they must be compared with the much smaller difficulties of any other special department of medicine. What are the difficulties, for example, of the aurist, or the oculist, or the dentist, in physiology, pathology, diagnosis and treatment, compared with the difficulties of him who takes the brain and the whole nervous system as his field of work? For compared with a mere nerve expansion like the retina, with its adjuncts, or the organ of Corti, with its necessary appendages, the whole brain is as the solar system to our little speck of earth.

The investigator of insanity looks to heredity, to nutrition, to function, and to accident, in the scientific pursuit of his work. And where else should he look? Where otherwise can he look? Does not the oculist, and the aurist, and the dentist look in the same directions, if he be a philosophical observer? If it be asked what is heredity, and how are its facts to be met and combatted? the answer is that heredity confers upon certain persons a nervous system which possesses certain variations from the normal standard. And is not this exactly what heredity sometimes does for the eye, the ear, the teeth, the muscles, the bones, the blood-vessels, and, indeed, any and every tissue and organ of the body? If, again, it be asked, how is such heredity as this to be combatted? the answer is, by judicious nutrition and function, and by bringing in the influence of a different heredity. And is not this precisely the answer which would be given if we were asked how we should combat the effect of heredity in cases of weak lungs, or of a tendency to rheumatism, or gout? In nothing does or can the brain differ from any other organ or tissue of the body except in the higher degree of its complexity, and in the consequent greater difficulties it presents to the physiologist, the pathologist, and the therapeutist.

If we are asked, Why, then, do we not prevent more insanity than we do? the answer is that the law, and the want of knowledge among the people, prevent us from getting patients under our control in those early periods of their insanity when their symptoms are as clear as noonday to the specialist, but entirely unperceived or misunderstood by the untaught and mostly self-confident and obstinate outsider. These are the reasons why we do not deal, and deal successfully, with the "earliest indications of danger." Other persons, unfortunately, deal with those "indications," and for the most part so foolishly as to convert temporary and harmless attacks of mental aberration into permanent conditions ofdistressing and dangerous insanity.

There is not space to pursue the subject further. But it ought to be pursued, that all classes among the people may know that in the department of lunacy, the same zeal, and the same scientific competence as characterise modern medicine generally are everywhere to be found.

